# Supplementary material for: Characterization of the Filovirus-Resistant Cell Line SH-SY5Y Reveals Redundant Role of Cell Surface Entry Factors
Source: Viruses. 2019 Mar 19;11(3):275. doi: 10.3390/v11030275 (PMC6466046; doi:10.3390/v11030275)
Supplement: Supplementary file 1 [file viruses-11-00275-s001.pdf]

SUPPLEMENTAL FIGURES:

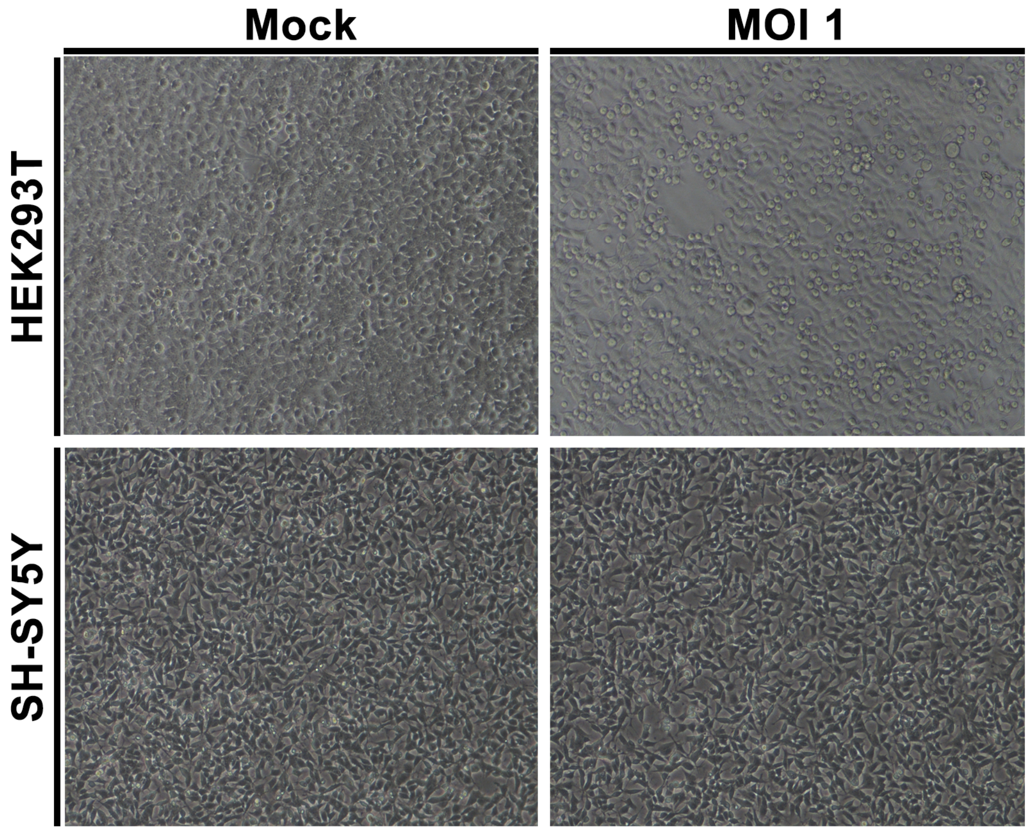

Figure S1. rVSVΔG-EBOV infection. Cell rounding caused by EBOV GP 24 h after mock or MOI 1 rVSVΔG-EBOV infection.

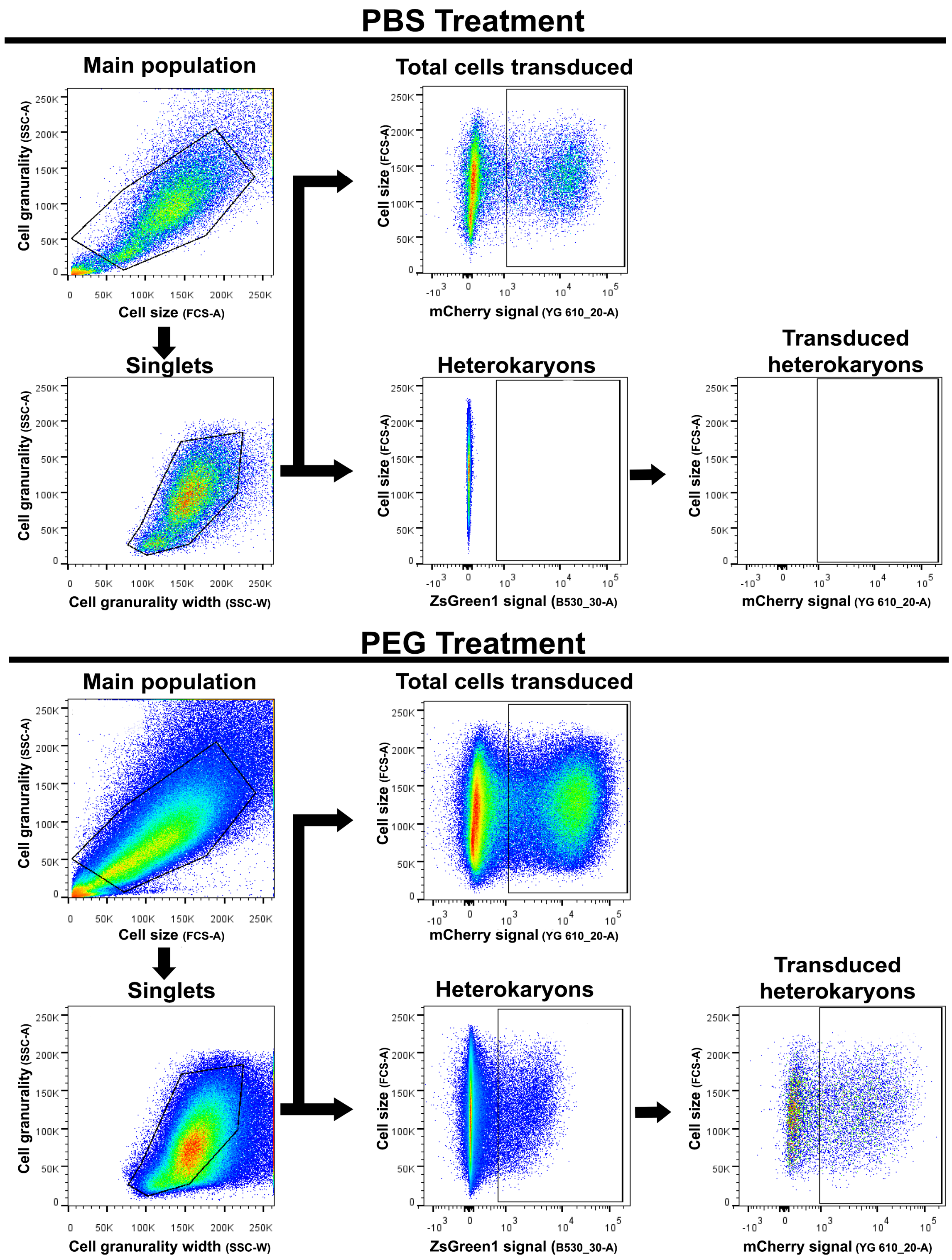

Figure S2. Gating strategy for detection of heterokaryons and transduced heterokaryons. First, cell main population was gated using cell size (FCS-A) over granularity (SSC-W). Then, single cells were discriminated by plotting SSC-A and SSC-W. For detection of total cell transduction (mCherry expression), YG 610_20A channel was used. For the detection of heterokaryons, ZsGreen1 expressing cells were gated on the B530_30-A green channel. To determine the percentage of transduced heterokaryons, ZsGreen1+ cells were drilled down and mCherry expression was determined using the YG 610_20A channel.

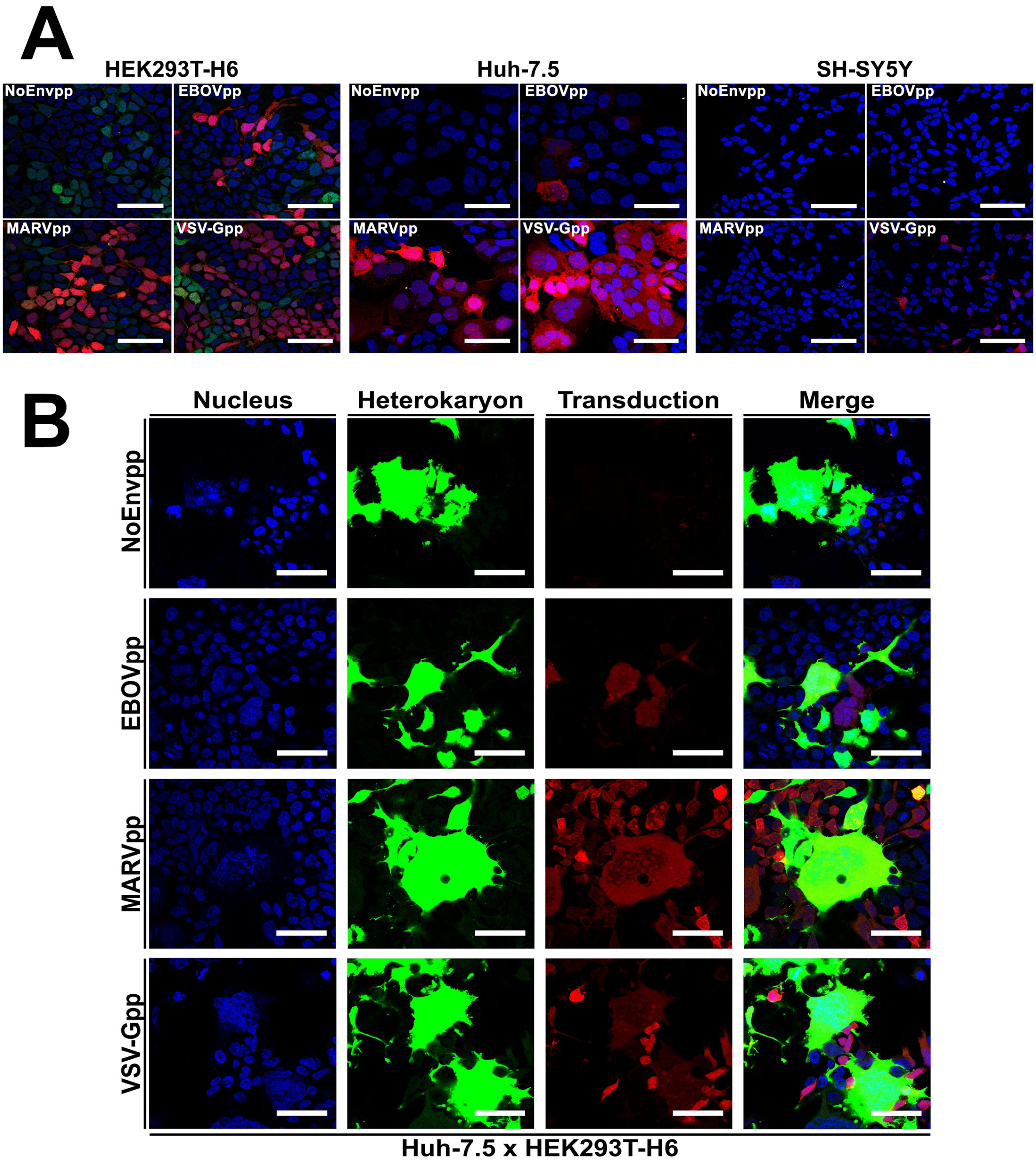

Figure S3. Susceptibility of single cell lines and heterokaryons of Huh-7.5 and HEK293T-H6 cells. (A) Single cell line transduction. Engineered cells for tetracycline inducible gene expression were transduced with the same NoEnvpp, EBOVpp, MARVpp, and VSV-Gpp / mCherry pseudoviral stocks as for Fig. 7 for 6 h at 37ºC and incubated for 72 h for mCherry expression (red). Cells were fixed with 3% PFA, permeabilized with 0.1% TX100, and stained with DAPI for nuclear staining (blue). Of note, HEK293T-H6 cells express low but detectable levels of ZsGreen1 but heterokaryons have a higher abundance of the protein allowing the differentiation of heterokaryons from background by microscopy and flow cytometry. Scale bars = 50 µm. (B) Susceptible Huh-7.5 and HEK293T-H6 cell fusion/transduction assay. Co-culture cells were fused with PEG for 5 min at 37ºC for heterokaryon formation (ZsGreen1 expression) and transduced 1 h later with the same viral stocks and methodology as above. Cells were fixed, permeabilized and stained as in (A).

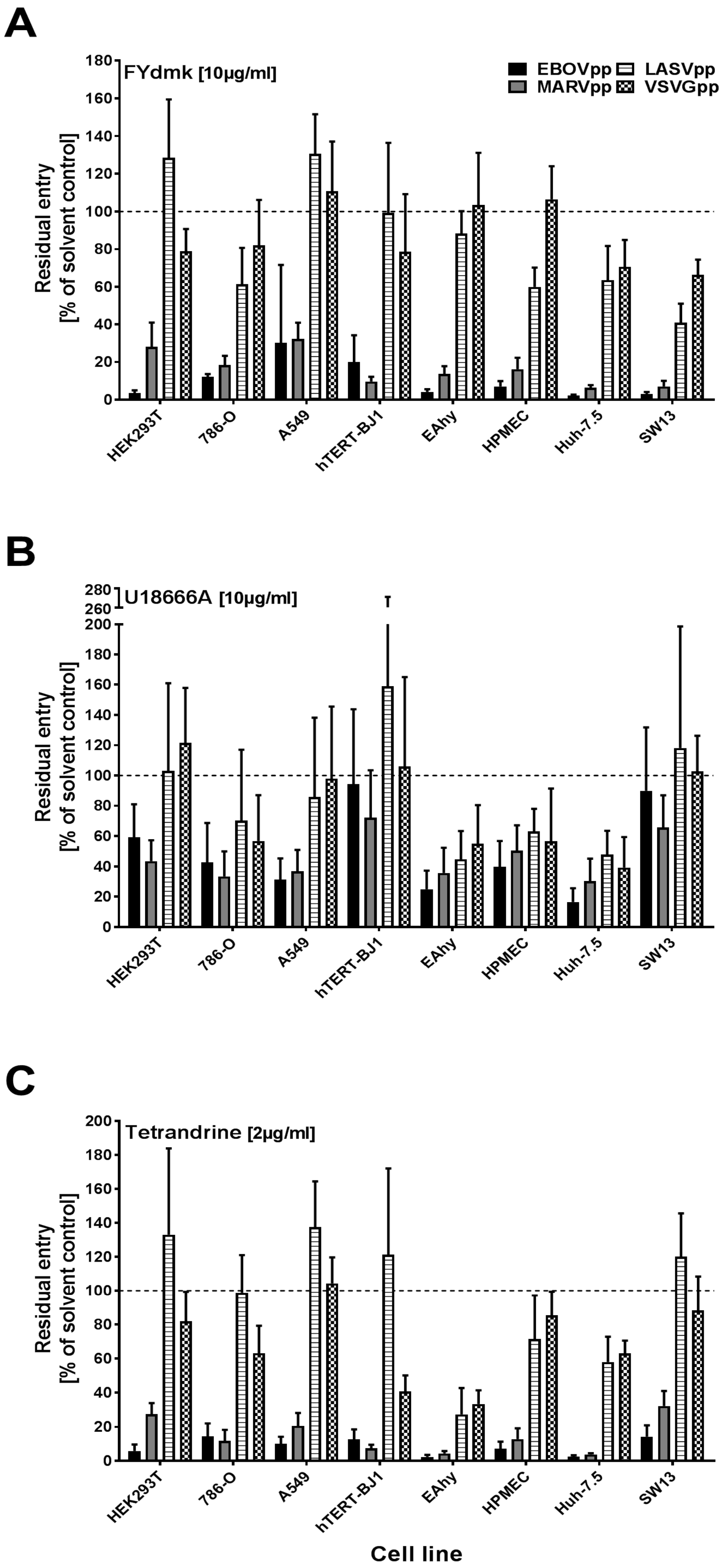
A
FYdmk [10µg/ml]
EBOVpp
MARVpp
LASVpp
VSVGpp
Residual entry
[% of solvent control]
HEK293T
786-O
A549
hTERT-BJ1
EAhy
HPMEC
Huh-7.5
SW13
B
U18666A [10µg/ml]
C
Tetrandrine [2µg/ml]
Cell line

Figure S4. Intracellular factors inhibition assays. Susceptible cell lines were treated with vehicle control or indicated drugs at the specified concentration 1 h prior to transduction. Subsequently, indicated pseudoparticles were added and further incubated for 6 h. After transduction, cells were washed with 1X PBS and incubated for another 72 h prior to luciferase activity measurement. Graphs are shown as the % residual entry after normalization to vehicle transduction. Each bar represents 9 individual values from 3 independent experiments each performed in triplicate with SD represented as error bars.

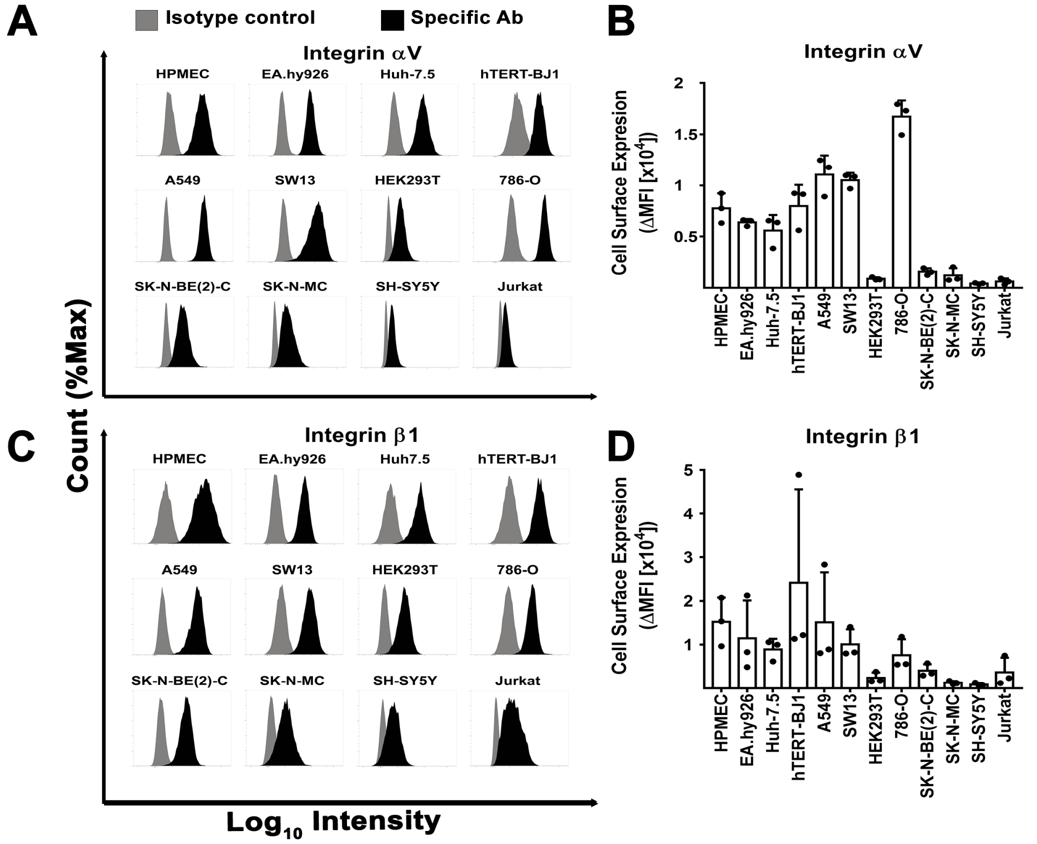

Figure S5. Integrin αV and β1 cell surface expression. Saturating concentrations of mAb against integrin αV (A-B), β1 (C-D) and respective isotype controls were used to detect surface protein levels of the indicated proteins in the panel of cell lines. Left-hand side represents histograms and right-hand side ΔMFI values. Graphs represent the mean of 3 independent stainings and ± SD.

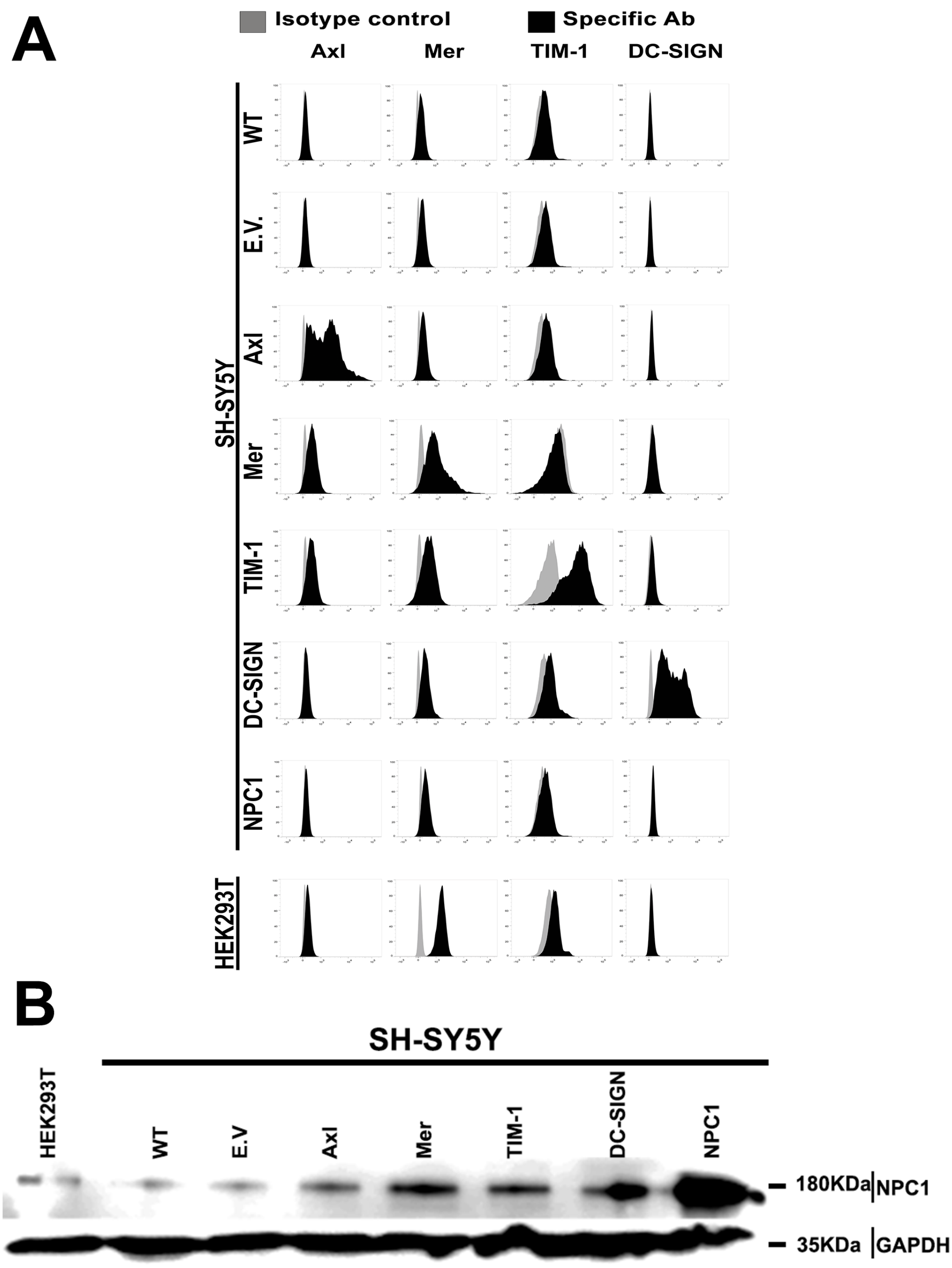

Figure S6. Detection of ectopic expression of filovirus attachment factors. (A) Cell surface staining of ectopically expressed surface factors. Saturating concentrations of APC-conjugated specific abs or isotype controls were incubated with live cells to determine the expression of the proteins at the plasma membrane. (B) NPC1 western-blot of HEK293T, SH-SY5Y WT and engineered cells.

TABLE S1. Reported filovirus attachment host factors.

Name	Gene Name	Protein family	Function in filovirus entry	Reference
**ASGR-1**	ASGR1	C-type Lectin	Interact with GP glycans	[26]

**ASGR-2**	ASGR2	C-type Lectin	Interact with GP glycans	[26]
**LSECtin**	CLEC4G	C-type Lectin	Interact with GP glycans	[22]
**DC-SIGNR**	CLEC4M	C-type Lectin	Interact with GP glycans	[24,25]
**DC-SIGN**	CD209	C-type Lectin	Interact with GP glycans	[24,25]
**hMGL**	CLEC10A	C-type Lectin	Interact with GP glycans	[20]
**FR-α**	FOLR1	Folate receptor	Unclear role in entry	[33,80]
**HPSG**	HSPG2	Proteoglycan	Attachment factor	[81]
**Ficolin-1**	FCN1	Ficolin family	Bridge GP to ficolin receptor	[82]
**Axl**	AXL	Tyrosine Kinase	Increased micropinocytosis	[35]
**Tyro3**	TYRO3	Tyrosine Kinase	Entry promotion, unclear	[29]
**Mer**	MERTK	Tyrosine Kinase	Entry promotion, unclear	[29]
**GAS-6**	GAS6	Soluble tk[a] ligand	Bridge envelope PS[b] to TAM tks	[83]*
**MFG-E8**	MFGE8	Soluble integrin ligand	Bridge envelope PS to integrins	[69]*
**TIM-1**	HVACR1	TIM	GP or envelope PS interaction	[28,64]
**TIM-4**	TIMD4	TIM	Envelope PS interaction	[27]
**Integrin αV**	ITGAV	Integrin	Regulate cathepsin activity	[31]
**Integrin β1**	ITGB1	Integrin	Regulate cathepsin activity	[31]
**SCARA1**	MSR1	Scavenger R. class A	PS related entry?	[56]
**SCARA2**	MARCO	Scavenger R. class A	PS related entry?	[56]
**CSR**	SCARA3	Scavenger R. class A	PS related entry?	[56]
**SCARA4**	COLEC12	Scavenger R. class A	PS related entry?	[56]
**NET33**	SCARA5	Scavenger R. class A	PS related entry?	[56]

a-tyrosine kinase; b-Phosphatidylserine;

*Studies performed with receptor binding domain deleted Sindbis E glycoprotein mutant viruses.

TABLE S2. Filovirus entry attachment factors microarray data (Relative Light Units)

	Cell line												
Gene Name	PHH	EA.hy926	Huh7.5	SH-SY5Y	SK-[illegible]	Jurkat	SK-N-SH	A549	786-[illegible]	hTERT-BJ1	HEK293T	HPMEC	SW13
ASGR1	284271	1072	17704	815	133	2767	350	248	31	212	166	80	87
ASGR2	197015	15	51317	20	16	20	18	27	22	20	19	22	22
CLEC4G	1425	21	15	15	15	15	15	16	17	22	15	20	20
CLEC4M	524	31	21	46	35	56	41	42	36	34	24	46	44
CD209	112	19	17	19	16	18	19	18	20	19	21	20	29
CLEC10A	191	18	214	19	15	17	19	29	17	16	19	15	22
FOLR1	220	60	98	106	290	69	1543	145	6776	743	166	66	341
HSPG2	503	22846	9454	434	523	192	4873	3878	12599	10170	430	3883	1284
FCN1	510	22	16	23	20	17	20	18	25	23	17	21	27
AXL	593	28006	61	72	142	50	12605	6730	1689[illegible]	41919	56	39230	102
TYRO3	58	1024	4129	2032	203[illegible]	1785	1194	1072	1588	1121	3423	596	3297
MERTK	652	758	897	156	133	79	198	85	60	15	302	71	484
GAS6	915	519	15	1593	15	15	869	222	2861	31132	295	1105	644
MFGE8	43	388	98	212	466	896	1185	482	683	2731	205	246	456
HAVCR1	20	130	976	15	15	15	15	513	1722	15	15	15	15
TIMD4	464	43	149	15	15	15	15	218	514	15	15	15	15
ITGAV	2119	10510	9994	2248	229[illegible]	924	6161	6446	13681	8923	1495	3958	13113
ITGB1	3362	6616	4000	2196	120[illegible]	917	5128	9686	9058	20496	513	12351	3764
MSR1	52	15	15	15	18	15	470	15	67	89	65	17	15
MARCO	6636	15	48	15	15	15	15	17	15	15	15	15	15
SCARA3	1235	2662	1966	989	576	194	3816	9760	1009	3323	430	13801	141
COLEC12	15	15	57	15	98	15	2295	15	15	658	675	308	15

**SCARA 5**	102	16	15	23	26	21	19	930	19	18	25	19	17
